# U-net based vortex detection in Bose–Einstein condensates with automatic correction for manually mislabeled data

**DOI:** 10.1038/s41598-023-48719-9

**Published:** 2023-12-02

**Authors:** Jing Ye, Yue Huang, Keyan Liu

**Affiliations:** Jiaxing Nanhu University, 572 Yuexiu South Road, Jiaxing, 314001 China

**Keywords:** Quantum simulation, Theoretical physics

## Abstract

Quantum vortices in Bose–Einstein condensates (BECs) are essential phenomena in condensed matter physics, and precisely locating their positions, especially the vortex core, is a precondition for studying their properties. With the rise of machine learning, there is a possibility to expedite the localization process and provide accurate predictions. However, traditional machine learning requires particular considerable amount of manual data annotation, leading to uncontrollable accuracy. In this paper, we utilize the U-Net method to detect vortex positions accurately at the pixel level and propose an Automatic Correction Labeling (ACL) approach to optimize the acquisition of data sets for vortex localization in BECs. This approach addresses inaccuracies in the labeled vortex positions and improves the accuracy of vortex localization, especially the vortex core positions, while enhancing the tolerance for human mislabeling. The main process involves Rough Labeling $\rightarrow$ Machine Learning $\rightarrow$ Probability Region Search $\rightarrow$ Data Relabeling $\rightarrow$ Machine Learning again. The objective of ACL is to secure more accurate labeled data for model retraining. Through vortex localization experiments conducted in a two-dimensional Bose-Einstein condensate, our results establish the following: 1. Even under conditions of biased and missing manual annotations, U-Net can still accurately locate vortex positions; 2. Vortices exhibit certain regularities, and training U-Net with a small number of samples yields excellent predictive consequences; 3. The machine learning vortex locator based on the ACL method effectively corrects errors in manually annotated data, significantly improving the model’s performance metrics, thus enhancing the precision and metrics of vortex localization. This substantial advancement in the application of machine learning in vortex localization provides an effective way for vortex dynamics localization. Furthermore, this method of obtaining more accurate positions of approximate human labels through machine learning offers new insights for machine learning in other types of image recognition problems.

## Introduction

Vortices are common features in fluid dynamics, present in various scientific fields such as fluid mechanics, superfluids, and astrophysics^1–4^. The experimental realization of Bose–Einstein condensates (BECs) provides an exceptional platform for exploring microscopic quantum phenomena^5,6^. BECs is a quantum state comprised of a group of bosons, formed at ultra-low temperatures, exhibiting precise coherence and low-energy excitations^7^. By combining artificial gauge field techniques, researchers can exert highly accurate control over BECs, simulating diverse quantum phenomena and gaining insights into its microscopic behavior^8–11^.

In BECs, quantized vortices represent one of the topological defect characteristics of superfluidity, arising due to the coherent nature of atomic wave functions within the BECs^5,12,13^. When BECs is limited in a rotating potential, vortices are formed, and their creation is directly proportional to the superfluid velocity and phase gradient of the order parameter^14,15^. Therefore, quantum vortices play a key role in understanding the behavior and properties of superfluids. Although quantum vortices have been extensively investigated in the field of superfluid helium, the experimental controllability of BECs has rekindled interest in investigating quantum vortices within it. Extensive and in-depth explorations have been made by researchers on various phenomena related to the formation, dynamics, and interactions of vortices in BECs^16–19^. Both in experimental and theoretical studies, it is necessary in order to accurately infer the precise positions of quantized vortices to delve into their dynamic behavior^20^. As vortices are topological defects within BECs with relatively complex internal structures, their accurate detection and localization pose challenging tasks. In experimental settings, methods for identifying and locating vortices in BECs often involve optical detection techniques. Optical detection techniques commonly employ imaging and scattering methods, where laser beams interact with BECs, allowing real-time monitoring of the morphology and position of vortices^21^. Theoretical methods, on the other hand, rely on establishing mathematical models built on the physical properties of BECs, and combining the characteristics of quantum vortices to infer their precise positions through data processing and simulation^22^. One significant challenge in accurately inferring vortex positions is their potential interference with other structures present in the system, such as weaknesses and vortices, which complicates the localization process. Consequently, enhancing the accuracy and precision of vortex localization remains a crucial issue in the current field of ultra cold atoms.

The rapid development in computer science and artificial intelligence has transformed machine learning algorithms into indispensable tools for investigating physical systems^23–25^. In comparison to traditional algorithms, the main advantage of machine learning resides in its ability to automatically learn features from data and reveal the underlying complex relationships in physical systems without relying on specific physical equations^26^. Recent research has shown significant advancements in using machine learning to solve the Schrödinger equation. By employing deep convolutional neural networks, it becomes possible to predict the ground state wave functions and energies corresponding to the Schrödinger equation accurately^27^. Subsequently, this idea was extended to more complex systems, such as multi-component and high-dimensional systems, utilizing various types of machine learning algorithms to achieve high-precision prediction of ground state wave functions and energy^28–33^, especially in complex systems where high-precision soliton solution prediction can be achieved^34^. These methods have achieved results beyond what habitual approaches could accomplish, offering new possibilities for experimental realization, understanding, and interpretation of quantum phenomena. Moreover, machine learning has relevant applications in generating, controlling, and detecting vortices^35^. Applying reinforcement learning techniques to control nonlinear matter waves, manipulating external Gaussian potentials to create and manipulate quantized vortices^36^, and utilizing convolutional neural networks to locate vortices and soliton positions in the density distribution images of BECs contribute to experimental and theoretical investigations of static and dynamic properties of vortex configurations and soliton characteristics in BECs^37,38^. The quality of the dataset is a crucial factor that determines the success of machine learning consequences. In the aforementioned machine learning case, manual data annotation is a difficult and time-consuming process. Therefore, optimizing this process and obtaining a higher quality dataset are important research directions.

In previous research, machine learning-based image recognition techniques were utilized to address the problem of vortex localization. These techniques involved dividing the original image into independent $4 \times 4$ grid cells and calculating the probability of a vortex core’s presence within each cell, along with determining the scaling factors (i.e., *x* and *y* coordinates) of the vortex core within that cell^37^. Guided by this work, in this paper, we conducted a supervised machine learning study on the localization of vortices in the ground state of a rotating BEC within a rotating frame. Our study adopted a more intuitive pixel-level U-Net model to obtain the probability associated with each pixel being part of the vortex core, effectively solving the vortex localization problem. Additionally, we proposed a novel automated approach to rectify potential errors in manually labeled data, reducing the time cost of manual vortex position annotation and improving the accuracy of labeling. This model is designed not only to detect vortices cores in density images, but also locate the exact pixel of the vortices cores’ centers. The goals of this study are (i) to analyze how accurately U-Net can predict vortex core center position, (ii) to estimate how many samples are needed to train the model for this regularly distributed density images, (iii) to check if and how well the model predicts when the manual labeling is not completely accurate, (iv) to propose a new Automatic Correction Labeling (ACL) method to solve the tedious manual labeling problem which is easy to make mistakes.

**Figure 1:**
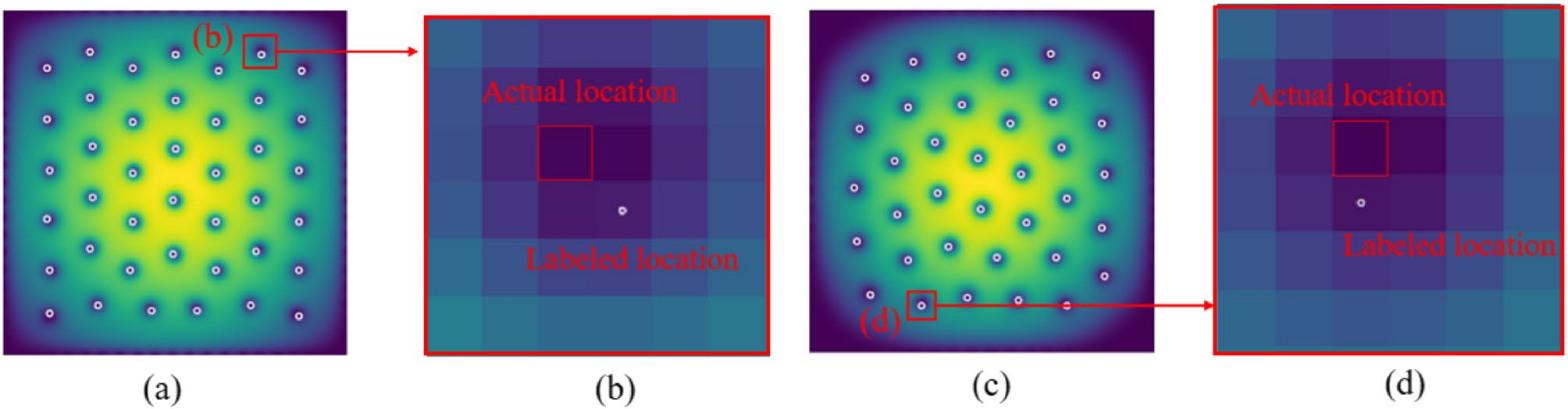
(**a,c**) Are the labeled images of vortex detection, in which white circles are the artificially labeled center points. (**b**) Is a magnification of the red rectangular marked area in (**a**), and (**d**) is a magnification of the red rectangular marked area in (**b**).

## Physical model

Consider a single-component BECs system made up of *N* atoms with mass *m*. These atoms are confined in the *z*-direction by a harmonic potential with frequency $\omega _z$ and in the perpendicular plane by a circular potential with radial frequency $\omega _\perp$ and radius ${\bar{r}} = \sqrt{{\bar{x}}^2+{\bar{y}}^2}$. With $\omega _z \gg \omega _\perp$, we obtain an approximate quasi-two-dimensional BECs system. To study the occurrence of vortices in the BECs, we apply a rotating potential, causing the system to turn around the *z*-axis with frequency ${\bar{\Omega }}$. At zero temperature and under the assumption of a dilute weakly interacting BECs, the system can be well-described within the mean-field theory framework. Therefore, the properties of the BECs can be described by a macroscopic wave function $\psi (x, t)$, and its dynamics follow the time-dependent Gross–Pitaevskii equation (GPE),

1 $$\begin{aligned} \begin{aligned} i\hbar \frac{\partial }{\partial t}\Psi =\left( -\frac{\hbar ^{2}}{2m}\nabla ^{2}+\frac{1}{2}m \omega _{\perp }^{2}{\bar{r}}^{2}+{\bar{g}}|\Psi |^{2}-{\bar{\Omega }} L_{z}\right) \Psi . \end{aligned} \end{aligned}$$

In the equation on the right-hand side, the terms are the kinetic energy, external potential, atomic interactions, and the contribution of the rotational field to the system, where $$ L_z=xp_y-yp_x$$ represents the angular momentum operator. The parameter $${\bar{g}}=4\pi \hbar ^2 a_s/m$$ denotes the strength of the interactions, which can be effectively adjusted through Feshbach resonance by changing the *s*-wave scattering length. For the convenience of numerical computations, we nondimensionalize Eq. (1) by taking $$\hbar \omega _\perp $$ as the energy scale and the corresponding time scale as $$1/\omega _\perp $$, with a length unit of $$\sqrt{\hbar /m\omega _\perp }$$. Thus, we arrive at the following dimensionless form of the two-dimensional GPE:

2 $$\begin{aligned} \begin{aligned} i\frac{\partial }{\partial t}\Psi =\left( -\frac{1}{2}\nabla ^{2}+\frac{1}{2}r^{2}+g|\Psi |^{2}-\Omega L_{z}\right) \Psi , \end{aligned} \end{aligned}$$

here, $g={\bar{g}}/\hbar \omega _\perp$, $\Omega ={\bar{\Omega }}/\hbar \omega _\perp$, and $r={\bar{r}}/\sqrt{\hbar /m\omega _\perp }$, are all nondimensionalized parameters. By solving Eq. (2), we obtain the ground state of the system for various parameters. Since Eq. (2) is a nonlinear equation that cannot be analytically solved, we employ imaginary-time evolution as the numerical approach to obtain the ground state of the system. In numerical simulations, our experimental setup is performed on a two-dimensional grid plane, representing the entire physical system. This grid consists of $2^7$ data points sampled in the $(x, y) \in [-2\pi , 2\pi ]$ space. The dynamical properties of the Bose-Einstein condensates (BECs) are governed by the GPE, with a given initial wave function $\psi _p = \exp {-(x^2+y^2)}$ as a Gaussian wave packet. The time iteration step for evolution is set to $10^{-3}$, and after $3 \times 10^6$ iterations, the ground state density distribution data of the system is achieved. To maintain the generality of the sample data, we randomly select parameters within the ranges of rotation frequency $\Omega =[0.6, 2.2]$ and interaction strength $g=[1000, 3000]$ for imaginary-time evolution to collect data. The above experimental settings can be extended to discrete systems, including multi-component BECs and various types of artificial gauge field configurations.

## Machine learning model

There are some reports in the literature on vortex detection in other related fields using supervised convolutional neural networks, especially U-Net achieves superior results^39,40^. In the following, we introduce the neural-network-based vortex detector motivated by U-Net which is designed for image segmentation to solve our private problems^41–44^. The overall task of image segmentation involves dividing an image into multiple segments or regions, where each segment represents a distinct object or area of interest^45^. Here, we are only interested in detecting vortices, and the sizes of vortices across the simulated images do not vary significantly, hence, we focus on predicting the exact position of each vortex core rather than the full vortex region. Therefore, our problem reduces to that of binary classification for each pixel, denoting the vortices cores by 1 and the other regions by 0. We used 456 images as the data set and manually labeled the vortices cores of them, 150 of which were used as the test data to calculate the metrics of the model and the rest as the training data.

In the process of machine learning, manual labeling data points consumes a significant amount of human labor and time^46,47^. Additionally, for this specific problem, the labeling process of denoting the vortices cores by 1 is very prone to errors due to human error. In the process of labeling, it is easy to deviate from the exact center position by labeling the neighboring pixels, and difficult to check. As shown in Fig. 1, the white circles are the artificially labeled vortices cores’ points, some of which we have zoomed in to show clearly. We zoom in red rectangular marked area (b) of (a), and red rectangular marked area (d) of (c). It can be found that the ideal center point is the pixel with the darkest color, but is actually labeled as the pixel near to it. This is a very common problem during the labeling process and can affect the training and output of the model, especially, it makes the metrics unreliable. Meanwhile, there will also be some vortex cores missing labeling.

**Figure 2:**
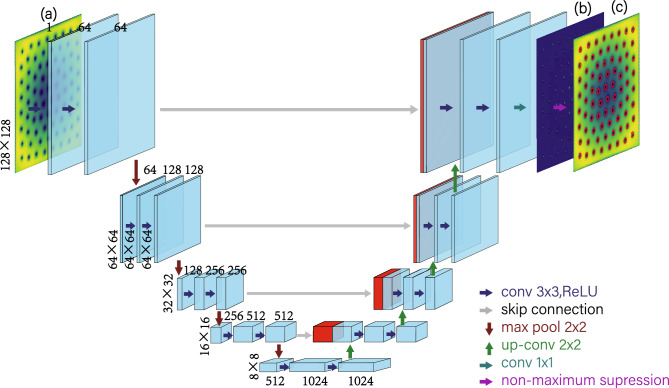
The network takes as its input images of dimension $128 \times 128$ with the condensate density (**a**), and outputs the probability of a vortex core in every pixel position (**b**). U-net architecture (example for 8 $\times$ 8 pixels in the lowest resolution). Each blue box is commensurate with a multi-channel feature map. The number of channels is denoted on top of the box. The *x*–*y*-size is offered to the lower left edge of the box. White boxes represent copied feature maps. The arrows denote the different operations.

Although the labeled data is not entirely correct, we use them directly for model training, and propose a new ACL method to solve the problem. The vortex detector model takes as its input gray scale images $I \in [0,1]^{W\times H \times C}$with equal width and height, $W = H = 128$, and a number of channels $C = 1$ to the U-Net neural network (see Fig. 2). In principle, the output of the detector can map a probability to each image pixel corresponding to whether this pixel represents a vortex core or not. The output *Y* of the neural network is therefore a tensor of dimensions $128 \times 128 \times 1$, where every number corresponds to the probability of a vortex core being attended by this pixel by applying Softmax function.

### Training model by the rough manually labeled data

Firstly, we train the model directly on density images obtained from simulations with the GPE, i.e., without the addition of any noise. Our training and testing data were acquired by manual labeling, which has not always been accurate in itself, as denoted in Fig. 1. The architecture of the model and the chosen hyper parameters are given in Fig. 2. The obtained density profiles are normalized such that their pixels lie between [0, 1] before being input to the neural network. We use the ADAM optimizer^48^ and a loss function of binary cross entropy with logits loss^49^. We have determined that giving a higher weight to learning positive predictions stabilizes training and accelerates convergence^50^, stabilizes training and accelerates convergence^50^.

**Figure 3:**
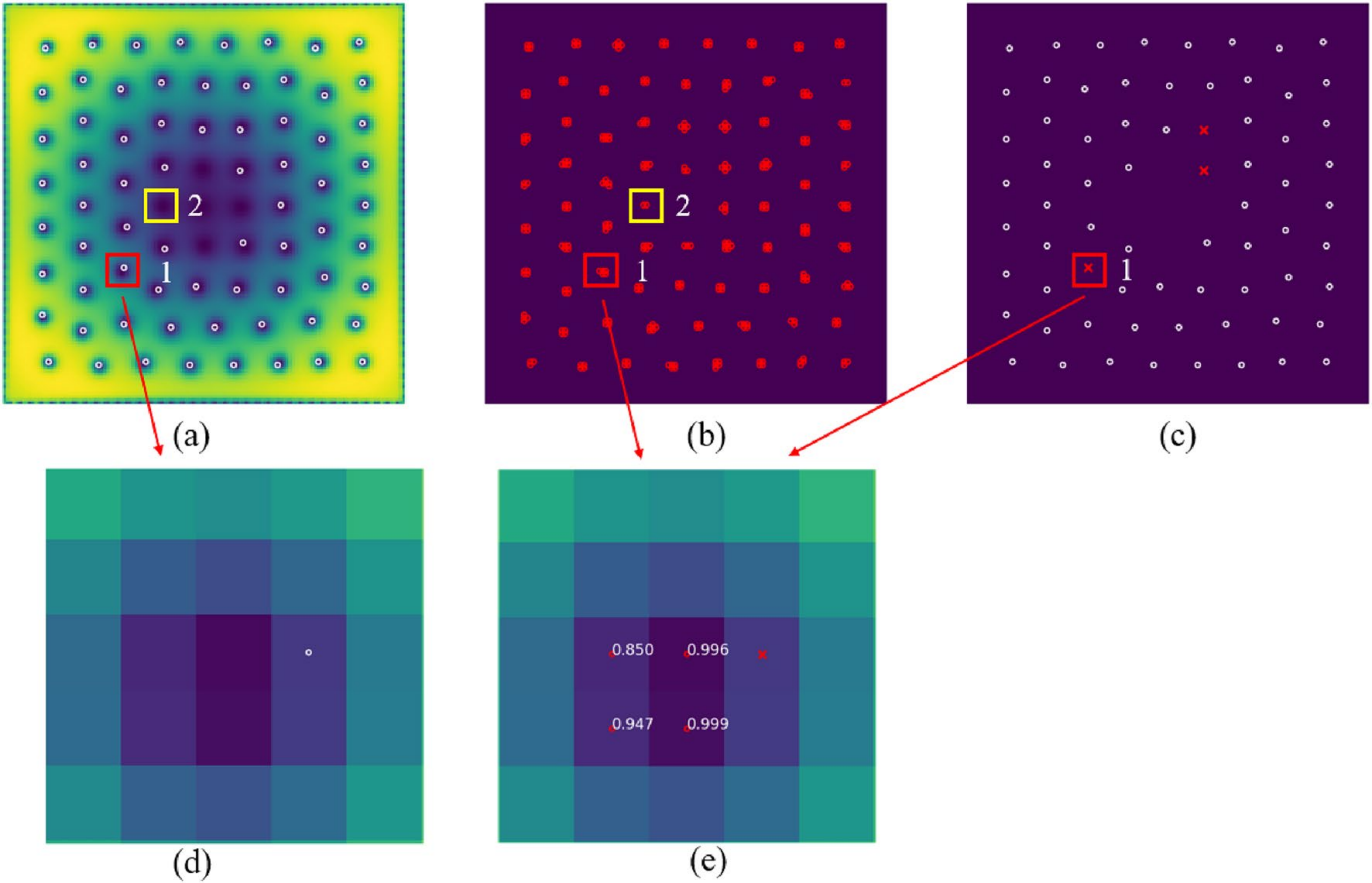
The locations of the vortex cores within each image are shown as red dots for the model prediction (**b**) and by white circles for the artificial labeling (**a**). In (**c**), the white circle means that the labeled points are among the predicted points, and the red cross means that the labeled points is not among the forecast points. (**d**) is a magnification of the red box 1 marked area in (**a**), and (**e**) is a magnification of the red box 1 marked area in (**b,c**). The numbers denoted in figure (**e**) is the predicted probability values.

Figure 3a,b show the same density image, where white circles in (a) correspond to the manual labeling and red dots in (b) to the prediction of the trained model. For the output probability tensor, the threshold is set to 0.5 in the experiment, where greater than 0.5 is 1 (detected as a vortex core center), and less than 0.5 is 0 (detected as not a vortex core center), as shown in Fig. 3. Overall, we achieved an accuracy value of 98.87% and a recall value of 99.57%, but only a precision value of 20.01% on the test data, as indicated in Table 1. Accuracy, precision and recall are calculated for every sample image through comparison between the position predicted by the model and the position labeled by manually, which is difficult to avoid making mistakes. According to our analysis, the main reason for high precision, high recall, and low precision is that the vortices cores are distributed sparsely on the density images, and even if none of the vortices cores are detected, such as all 0 (less than threshold 0.5), the accuracy will also be high, as shown in Fig. 3a. More importantly, we can find that some results detected by the model are very close to the actual vortices cores centers though some of the labeled data has errors. Moreover, the vortices cores with missing labels are automatically detected, as shown with the yellow box denoted by the yellow box denoted by 2 in Fig. 3b. According to the results provided for in the model, most of the vortices cores were detected, but many ambiguous pixels were also detected around the ground truth. The number of detected vortices cores is much greater than the actual number, as shown in Fig. 3b, which leads to the low precision. In Fig. 3c, the white circles indicate that the labeled pixels are among the detected results, and the red cross indicates that the labeled pixels are not among the detected results. Figure 3d,e show the magnification of the red box area denoted by 1 in Fig. 3a,b, respectively, and the numbers in Fig. 3e show the probability of the output result $(>0.5)$. In Fig. 3e, we can find that the pixel with the highest probability of the model detected result is just the true vertex core position, but the labeled pixel is the pixel near the ground truth, which is not detected by the model. Therefore, we adjust the position of the labeled pixel to the maximum probability of the detected results, and reliable the data set for training and testing.

### Training model by the ACL iteration data

**Figure 4:**
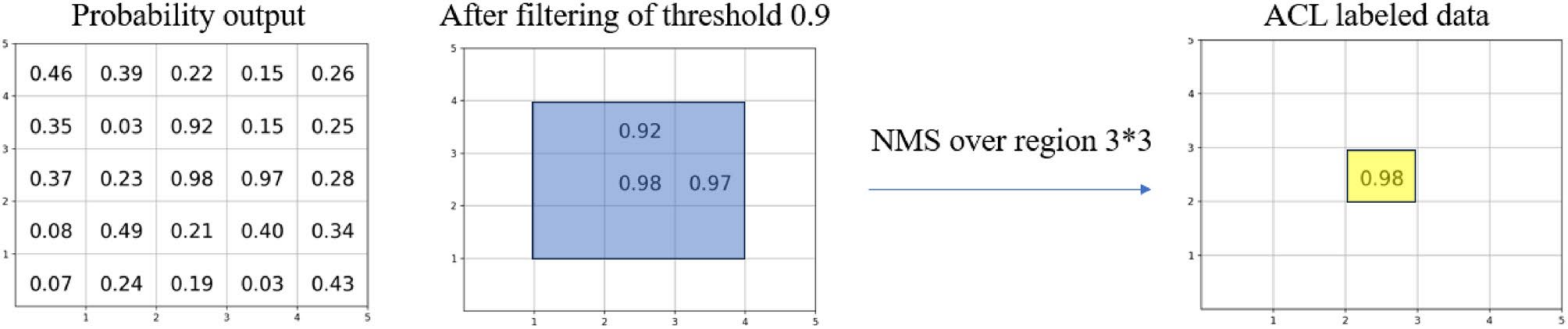
Schematic diagram of the pseudo processing of ACL, in which the threshold is 0.9 and the NMS window size is $3 \times 3$, as the blue box shown.

We use a new ACL method to relate the vortices cores position. After setting the threshold value (0.9) to the output of the model, by which the probability values less than 0.9 are reduced to 0, as times the mask. Then, non-maximum suppression (NMS) method^51,52^ is used to remove neighbouring ambiguous pixels detected. We create a window centered on the pixel position of the maximum probability, and suppress all other pixels in the window to 0, so as to iterate this process to all nonzero values in each image sample. In Eq. (3), $I_{i-1}$ represents the input density image to be detected, *NN* represents the neural network function, *Mask* represents the thresholding operation, *NMS* represents the non-maximum suppression operation, B represents the binary classification operation, and $L_i$ represents the labeling data by ACL, in which *i* represents the iteration time. The pseudo processing of ACL is shown in the Fig. 4, in which we threshold all values below than 0.9 and use the NMS window size of $3 \times 3$. Finally, the yellow pixel position is labeled as the new vortex core position.

3 $$\begin{aligned} {\mathscr {L}}_{i}=B(NMS(NN(I_{i-1}) \times Mask)). \end{aligned}$$

**Figure 5:**
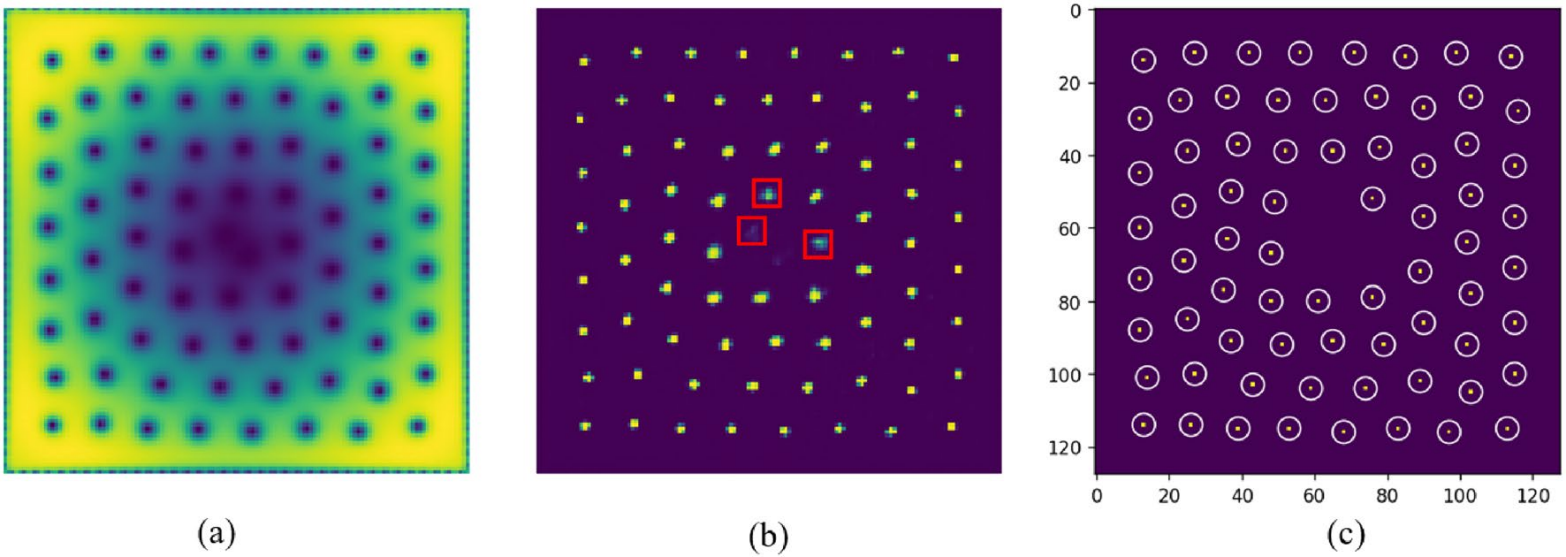
(**a**) is the input image of the condensate density. (**b**) is the probability output of the model by threshold over 0.5, in which red boxes indicate ambiguous points. (**c**) is the labeled data using ACL.

In the actual experiment, we choose the size of window of MNS in our data set to be $5 \times 5$, and thus ensure that the size is chosen sufficiently fine that at most one vortex core is present in any window, as shown in Fig. 5. From the experimental results, the ACL method can automatically adjust the position of labeled vortex cores position, and it’s more accurate than the manual labeling for exact pixels.

The probability output of the model with the threshold of 0.5 has many ambiguous points around the exact vortices cores position, as shown in Fig. 5b. The red boxes in Fig. 5 represent the probabilities of points are greater than 0.5, but we want to remove them. So in ACL, we set the threshold value to 0.9. Then, to do away with the neighbouring ambiguous points, the NMS method is also used for the output result. The relabeled vortices cores are denoted by white circles in Fig. 5c.

The performance of the model is measured by four metrics, namely Accuracy (${\mathscr {A}}$), Precision (${\mathscr {P}}$), Recall ($\mathscr {R}$) and F1-score (F1). The F1-score is the harmonic mean of the recall and precision, and these metrics are defined as follows:

4 $$\begin{aligned} {\mathscr {A}} =\frac{\textrm{TP}}{\mathrm {TP+FP+}TN+FN},\quad {\mathscr {P}}=\frac{\textrm{TP}}{\mathrm {TP+FP}},\quad {\mathscr {R}}=\frac{\textrm{TP}}{\mathrm {TP+FN}},\quad \textrm{Fl}=\frac{2\times \mathrm {P\times R}}{\mathrm {P+R}}, \end{aligned}$$

where $\textrm{TP}$, $\textrm{FP}$, $\textrm{TN}$, and $\textrm{FN}$ denote the number of true positives, false positives, true negatives, and false negatives, respectively, in all of the test samples, where each sample has $128 \times 128$ pixels to calculate. These four values all ranges from 0 to 1, with a higher value denoting more accurate detection. After using the NMS method to the predicted probability outcome, the precision and $\textrm{F1}$ score value are dramatically increased while the recall value is somewhat decreased, as shown in Table 1.

**Table 1:** Detector performance metrics (accuracy, precision, recall and F1 score) are computed on the test data using manual labeling and ACLs.

Detection	Accuracy $({\mathscr {A}})$ (%)	Precision $({\mathscr {P}})$ (%)	Recall $({\mathscr {R}}) (\%)$	F1 score (%)
Using manual labeling	98.87	20.01	99.57	33.31
Using manual labeling after NMS	99.81	65.93	66.84	66.36
Using ACL $1^{st}$ time	99.69	49.33	99.97	65.83
Using ACL $1^{st}$ time after NMS	99.92	85.64	86.28	85.95
Using ACL $4^{th}$ time	99.78	59.28	99.97	74.08
Using ACL $4^{th}$ time after NMS	99.97	94.02	95.31	94.64

In the case of ACL 4th time, it is calculated using the relabeled data iterated from the 3rd time ACL.

**Figure 6:**
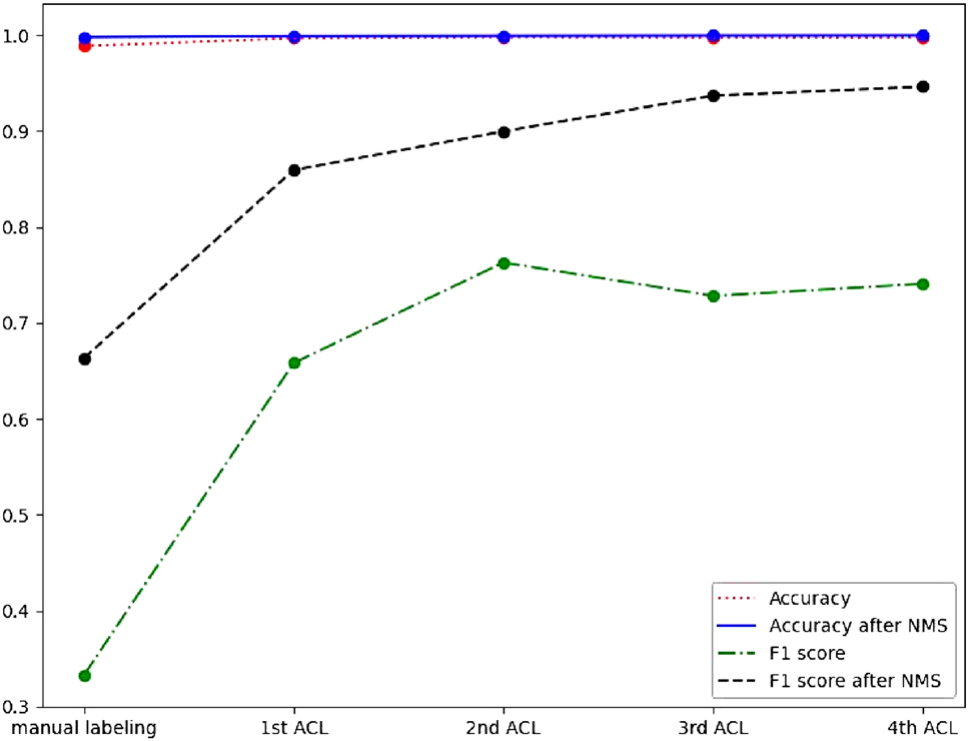
Accuracy and F1 score versus manual labeling and iterative ACLs for prediction output by thresholding only and by both thresholding and NMS.

To improve the performance of the model and make the metrics more accurately represent the model results, we use the labeled data by ACL and retrain the model. Then we carry out a second ACL on the labeled data from the first ACL ($i=2$ in Eq. 3). The relabeled data from the second ACL is then used, and the retained model is used to perform a third ACL on the data from the second ACL, and so on. The general process is: rough labeling $\rightarrow$ machine learning $\rightarrow$ probability region searching $\rightarrow$ relabeling the data $\rightarrow$ machine learning again. In Table 1, all the metrics are improved after ACL are iterated four times. The prediction metrics of both F1 score and accuracy by using manual labeling and ACL are gradually improved with the number of iterations, as shown in Fig. 6. Figure 6 shows that the first use of ACL improved the metrics dramatically, and then improved slowly after each iteration. We also find that the increase of ACL iteration times can improve accuracy slightly and F1 score greatly, especially F1 score after NMS, which is our detection method in practice, as shown by the black dashed line in Fig. 6. NMS is essential for this peculiar vortex core detection problem, because the pixels around the ground truth position also detect as high probability values, which are hard to judge directly with a threshold value.

**Figure 7:**
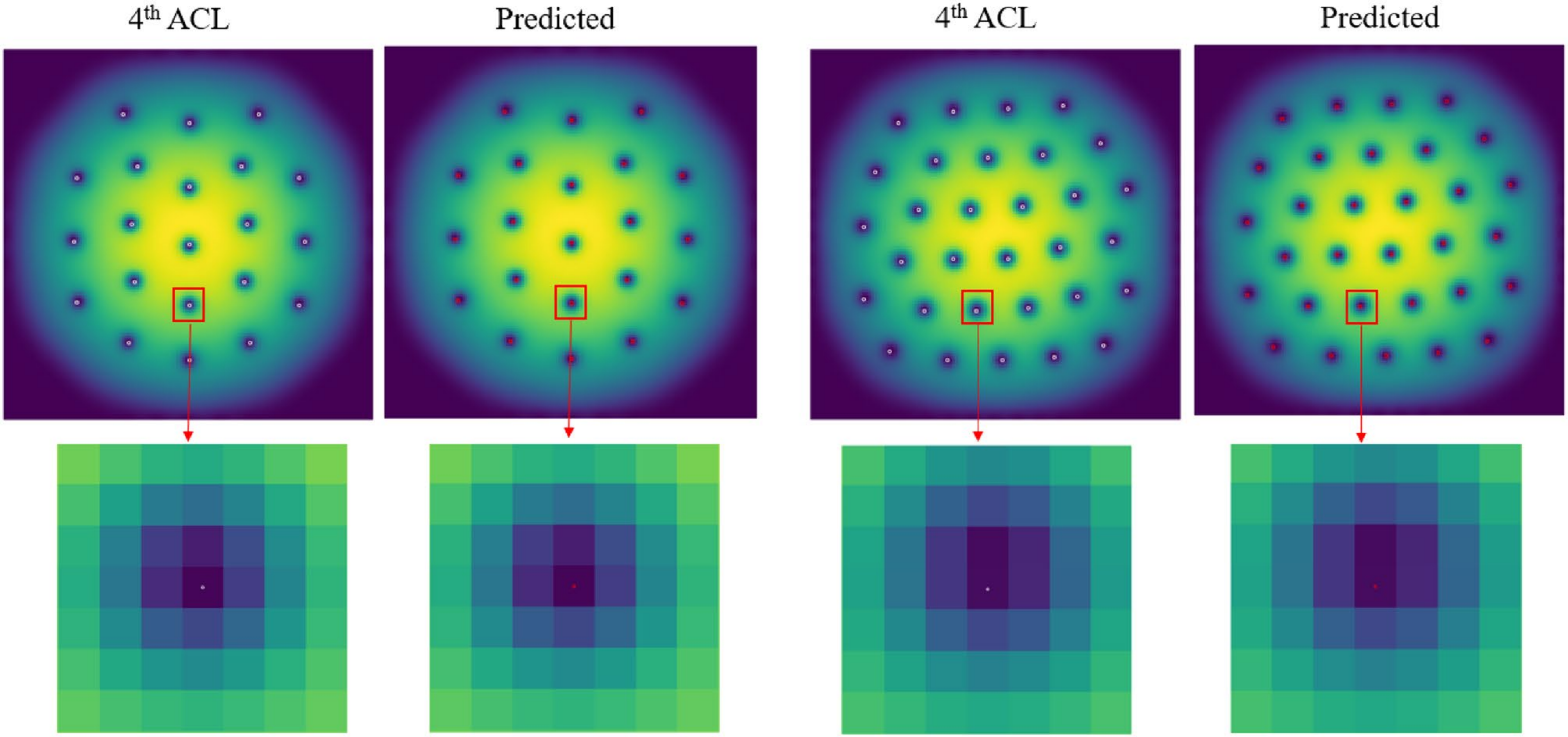
4th ACL labeled images and predicted output of the images, in which white circles are the labeled position of vortex cores and red circles are the predicted position by the model.

To compare with the observed problems in Fig. 1, the labeled regions using 4th ACL and the model predictions (using NMS) were observed in magnification, as shown in Fig. 7. We randomly selected 2 test samples and the zoomed in the area around the labeled position. From the magnification area of the red box, it can be seen that the 4th ACL labeled position and the predicted position all have been located at the ground truths.

**Figure 8:**
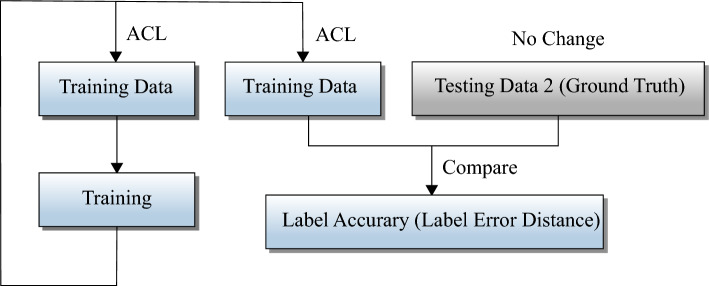
Structure block diagram of calculation method of label accuracy.

In order to demonstrate that ACL method can indeed correct false manual labeling, we use label accuracy and label error distance to compare before and after ACL with ground truth labels. The calculation flow is shown in Fig. 8. Label accuracy (LA) is the percentage of the altered labels over the ground truth labels, which is as defined as follows:

5 $$\begin{aligned} LA = \frac{1}{N} \sum _{i=1}^{N}\left( \sum _{j=1}^{S} \frac{I_j}{S}\right) , \end{aligned}$$

where *N* denotes the number of test samples, *S* denotes the number of vortex core on the *i*th sample, $I_j$ is 0 if the label point is not matched with the ground truth label point or 1 if the label point is matched with the ground truth label point. Obviously, this LA metric can calculate the effectiveness of using the ACL method to adjust the position of the label points.

We also use another metric label error distance (LED) to observe the change of labeling correction with ACL method, which is defined as follows:

6 $$\begin{aligned} LED = \sum _{i=1}^N \frac{ d_1+d_2+ \cdots +d_S}{S}, \end{aligned}$$

where *N* denotes the number of test samples, *S* denotes the number of vortex core on the *i*th sample, $d_j$ is the distance between the label point and the ground truth label point in pixels.

It can be obtained from the calculation of Eq. (5) that the metrics of LA is the higher the better, indicating that the more labels are matched with the ground truth labels. On the other side, label error distance (LED) is the lower the better, indicating that the pixel position of labels is closer to the ground truth labels’ pixel position. From Table 2, we can see that the accuracy of the labeling has been greatly improved by the ACL method, from 65.89% to 83.44%, which is improved greatly. It is worth noting that the second iteration of ACL results in a decrease in LA value, although it still higher than before using ACL. And the values of LA barely change as we continue to iterate. The results of this experiment are different from those obtained by the previous iterative ACL method. In addition, the value of LA and the value of LED are inversely correlated, and we can get the same conclusion from the value of LED in the Table 2.

**Table 2:** Label accuracy (LA) and label error distance (LED) stands for the effectiveness of the ACL method.

	Label accuracy (LA) (%)	Label error distance (LED)	F1 using ground truth labels (%)
Comparing with manual labeling	65.89	2913.14	83.01
Comparing with ACL 1st time	83.44	1355.79	86.36
Comparing with ACL 2nd time	76.42	1887.31	84.40
Comparing with ACL 3rd time	76.42	1887.31	85.94
Comparing with ACL 4th time	76.15	1980.61	86.23

F1 scores using ground truth labels show that the ACL method can be used to correct false manual labeling.

Furthermore, we use the trained model and ground truth label data to calculate the F1 score. From Table 1, the F1 score is only 66.36% by using the manual mislabeled data, compared with the F1 score of 83.01% by using the ground truth labels in Table 2. This can prove that even if the manual labeling is not completely correct, these data can still be used to train the U-Net and get good detection results, which is a feature of the U-Net network that has not yet been proved by the data, as far as we know. The F1 score with ground truth labeling has also been improved by the ACL method, from 83.01 to 86.36%. But it is decreased with the second iterative ACL, and the F1 score increases slightly with the number of iterations, but it is still less than when the ACL was first used. This differs from Fig. 6, where the F1 score increases as the number of iterations of ACL increases. This means that with this iterative ACL method, labels agree with whatever the U-Net model favors. Therefore, in practical application, it is meaningful to use this ACL method for the first time, not only to adjust the manual mislabeled data, but also to get a higher metrics score based on the adjusted labeling data.

In order to establish appropriate uncertainties and demonstrate the generality of the ACL method, we artificially created 1000 data samples and experimented with fivefold cross-validation. Each sample is an image of $128 \times 128$ pixels, on which *N* $(1<N<36)$ partially overlapping two-dimensional Gaussian functions are randomly superimposed. The purpose of the experiment is to accurately detect the vertex positions of all Gaussian functions. First, we randomize the positions of vertices in the training data set (800 samples) to simulate human mislabeling data, and use the model in Fig. 2 to train the training data set and calculate the evaluation metrics on the test data set (200 samples), which vertex positions are ground truth. Then we run the ACL method using the trained model, and re-train a new model to compare the results of evaluation metrics with the pre-trained model. We repeated the experiment five times in the cross-validation and obtained the results as shown in Table 3.

**Table 3:** The comparison of the evaluation metrics (precision, recall and F1 score) between the model trained on the original data of artificial randomized superposition of 2D Gaussian functions and the model trained after ACL adjustment in the fivefold cross-validation.

	Precision		Recall		F1 score	
	Original data (%)	Using ACL (%)	Original data (%)	Using ACL (%)	Original data (%)	Using ACL (%)
Experiment 1	71.89	74.06	72.54	74.06	72.17	74.06
Experiment 2	96.0	99.95	96.0	99.95	96.0	99.95
Experiment 3	75.94	99.09	97.23	99.09	82.33	99.09
Experiment 4	65.62	71.27	65.67	71.27	65.64	71.27
Experiment 5	63.81	68.20	63.81	64.55	63.81	65.99
Average result	74.65	82.51	79.05	81.78	75.99	82.07

From all the above experiments, it is concluded that this ACL method can improve the performance of vertex position detection model during training, in the case of training data is not very accurate. From the data of Table 3, we can also find a trend that when the evaluation metrics of the original model is better, the ACL method is more effective in improving the model’s performance. For example, in experiment 3, the F1 score was increased from 82.33 to 99.09% by using ACL.

## Conclusion

In summary, this study is built on machine learning techniques and aims to locate vortices in two-dimensional BECs. To improve the correctness of manually labeled data, we have proposed a novel automatic labeling method based on machine learning called Vortex Core Detection ACL. This method applies an machine learning based approach to automatically correct errors in manual annotations. Furthermore, we are about to explore the application of this automatic labeling technique to other aspects of ultra cold atoms, such as machine learning-based localization of solitons. The experimental results demonstrate that the ACL method effectively corrects errors in manually labeled data, from 65.89 of LA to 83.44% of LA, and significantly enhances the model’s performance metrics, such as F1 score. However, as the number of ACL iterations increases, the performance of the ACL method has a tendency to stabilize and no longer exhibits further improvement on relabeling data. Additionally, it is essential to note that labeling have a substantial impact on the training and detection results of the model. A crucial issue addressed by ACL is accurately labeling the pixels surrounding the vortex core at the exact center. In this method, the threshold value is a critical hyperparameter, which we set to 0.9 during experiments. If the threshold value is adjusted too high, it may miss the ground truth positions, while setting it too low can lead to erroneous labeling of ambiguous points. Moreover, the size of the NMS window in ACL is another important hyperparameter, and we set it to 5 $\times$ 5 in our experiments. The reason for its significance is comparable to that of the threshold value. Building on existing research, we further apply the ACL method to address mislabeled data related to U-Net, and find that U-Net can still detect the exact positions of vortex cores if the manual labeling is not very accurate. Last but not least, we discovered the potential of U-Net in automatically correcting vortex cores in mislabeled data, which is of significant importance for advancing the application of machine learning in ultra cold atoms.

## Data Availability

The raw and processed data for this work can be obtained from Ref.^53^, and the computer code can be obtained from the corresponding author upon reasonable request.
